# Twin robotic x-ray system in small bone and joint trauma: impact of cone-beam computed tomography on treatment decisions

**DOI:** 10.1007/s00330-020-07563-5

**Published:** 2020-12-05

**Authors:** Jan-Peter Grunz, Lenhard Pennig, Tabea Fieber, Carsten Herbert Gietzen, Julius Frederik Heidenreich, Henner Huflage, Philipp Gruschwitz, Philipp Josef Kuhl, Bernhard Petritsch, Aleksander Kosmala, Thorsten Alexander Bley, Tobias Gassenmaier

**Affiliations:** 1 Department of Diagnostic and Interventional Radiology, University Hospital Würzburg, Oberdürrbacher Straße 6, 97080 Würzburg, Germany; 2 Institute for Diagnostic and Interventional Radiology, Faculty of Medicine and University Hospital Cologne, University of Cologne, Kerpener Straße 62, 50937 Cologne, Germany; 3 Department of Trauma, Hand, Plastic and Reconstructive Surgery, University Hospital Würzburg, Oberdürrbacher Straße 6, 97080 Würzburg, Germany

**Keywords:** Cone-beam computed tomography, Extremities, Fractures, bone, Radiography

## Abstract

**Objectives:**

Trauma evaluation of extremities can be challenging in conventional radiography. A multi-use x-ray system with cone-beam CT (CBCT) option facilitates ancillary 3-D imaging without repositioning. We assessed the clinical value of CBCT scans by analyzing the influence of additional findings on therapy.

**Methods:**

Ninety-two patients underwent radiography and subsequent CBCT imaging with the twin robotic scanner (76 wrist/hand/finger and 16 ankle/foot/toe trauma scans). Reports by on-call radiologists before and after CBCT were compared regarding fracture detection, joint affliction, comminuted injuries, and diagnostic confidence. An orthopedic surgeon recommended therapy based on reported findings. Surgical reports (*N* = 52) and clinical follow-up (*N* = 85) were used as reference standard.

**Results:**

CBCT detected more fractures (83/64 of 85), joint involvements (69/53 of 71), and multi-fragment situations (68/50 of 70) than radiography (all *p < 0.001*). Six fractures suspected in radiographs were ruled out by CBCT. Treatment changes based on additional information from CBCT were recommended in 29 patients (31.5%). While agreement between advised therapy before CBCT and actual treatment was moderate (*κ* = 0.41 [95% confidence interval 0.35–0.47]; *p < 0.001*), agreement after CBCT was almost perfect (*κ* = 0.88 [0.83–0.93]; *p < 0.001*). Diagnostic confidence increased considerably for CBCT studies (*p < 0.001*). Median effective dose for CBCT was 4.3 μSv [3.3–5.3 μSv] compared to 0.2 μSv [0.1–0.2 μSv] for radiography.

**Conclusions:**

CBCT provides advantages for the evaluation of acute small bone and joint trauma by detecting and excluding extremity fractures and fracture-related findings more reliably than radiographs. Additional findings induced therapy change in one third of patients, suggesting substantial clinical impact.

**Key Points:**

*• With cone-beam CT, extremity fractures and fracture-related findings can be detected and ruled out more reliably than with conventional radiography.*

*• Additional diagnostic information provided by cone-beam CT scans has substantial impact on therapy in small bone and joint trauma.*

*• For distal extremity injury assessment, one-stop-shop imaging without repositioning is feasible with the twin robotic x-ray system.*

## Introduction

Evaluation of extremity trauma is among the most common imaging tasks in any emergency department [1]. While digital radiography remains the primary means of fracture diagnosis due to cost-efficiency, low radiation dose, and ubiquitous availability, computed tomography (CT) can provide advantages over x-ray imaging in detection of subtle fractures or visualization of fracture displacement and assist pre-surgical planning [2–4]. Furthermore, cone-beam CT (CBCT) can contribute to trauma assessment by ruling out fractures that were suspected in previous radiography [5–7]. The main disadvantage of CT imaging lies in the increased radiation dose compared to conventional x-ray scans [8]. However, ongoing technological advances in scanner and detector construction nowadays facilitate low-dose studies with diagnostic image quality [9–11].

For clinical CT imaging, two types of image acquisition are mainly incorporated in today’s scanners. Multi-detector CT (MDCT) systems feature fan-shaped x-ray beams and multiple detector rows that rotate around the patient. Table movement facilitates large scan volumes through helical image acquisition. Remaining closer to its radiographical roots, CBCT systems rely on flat-panel detectors and pyramid-shaped beam geometry. Without helical movement, their field of view (FOV) is limited by the detector size, yet superior spatial resolution provides potential benefits for extremity imaging [12, 13]. After superseding MDCT as the imaging standard for maxillofacial surgery and dentistry in the 1980s and 1990s, CBCT has also gained recognition for the appendicular skeleton during the last decade [14–17]. With the emergence of dedicated extremity scanners, the combination of flexible positioning, weight-bearing options, decreased radiation dose, and first-rate image quality led to CBCT being increasingly used for trauma imaging [18–20]. Despite their advantages, one point of criticism towards CBCT scanners remains the lack of versatility compared to MDCT systems due to restrictions of scanner architecture (e.g., small source-to-image distance, narrow FOV).

The twin robotic x-ray system used in this study overcomes these limitations through its open construction and large flat-panel detector. Being capable of radiography, fluoroscopy, and CBCT of most body parts, the scanner appears promising as a multi-use tool for trauma imaging. Recent studies were able to show potential for considerable dose reduction, when comparing the CBCT scan mode to MDCT in cadaveric specimens [21, 22] and patients [23]. Literature analyzing the actual impact on treatment in acute trauma of the appendicular skeleton is lacking though. Hence, the aim of this work was to investigate the added clinical value of the multi-use system’s CBCT scan mode for extremity fracture assessment in patient studies, compared to conventional radiography.

## Material and methods

### Study participants

**Fig. 1:**
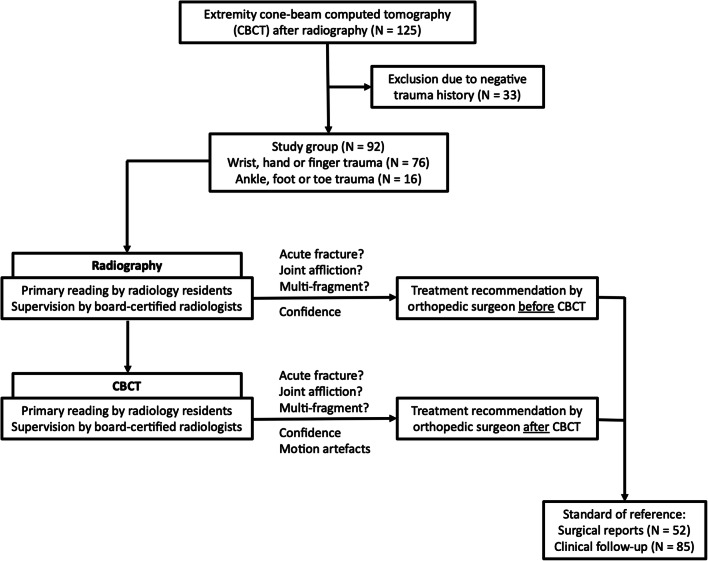
Flow chart for visualization of study inclusion/exclusion criteria, study population, and individual steps of the data analysis

For this retrospective study, informed consent was waived, and permission obtained from the local Institutional Review Board. This work was conducted in accordance with institutional and national research committee requirements as well as the 1975 Declaration of Helsinki. Between January and June 2020, 125 consecutive patients underwent extremity cone-beam CT imaging after digital radiography at our department. Acute trauma history was mandatory for study inclusion; hence, 33 patients had to be excluded. The final population consisted of 92 patients (42 women, 50 men; mean age 48.7 ± 19.4 years; range 18–87 years). Wrist, carpal, or finger trauma was reported in 76 cases (40 left, 36 right). Ankle or foot trauma history was positive in 16 patients (11 left, 5 right). Patients were allocated to CBCT after radiography either to provide 3-D imaging for surgical planning (in displaced or presumably articular fractures) or for detection/verification/exclusion of radiographically occult/questionable fractures. Imaging tasks included detection and classification of upper and lower extremity fractures, as well as in-depth analysis of fracture-related findings like joint affliction and multi-fragment situations. Figure 1 shows a flow chart summarizing inclusion and exclusion criteria and the final study group.

### Image acquisition and reconstruction

**Fig. 2:**
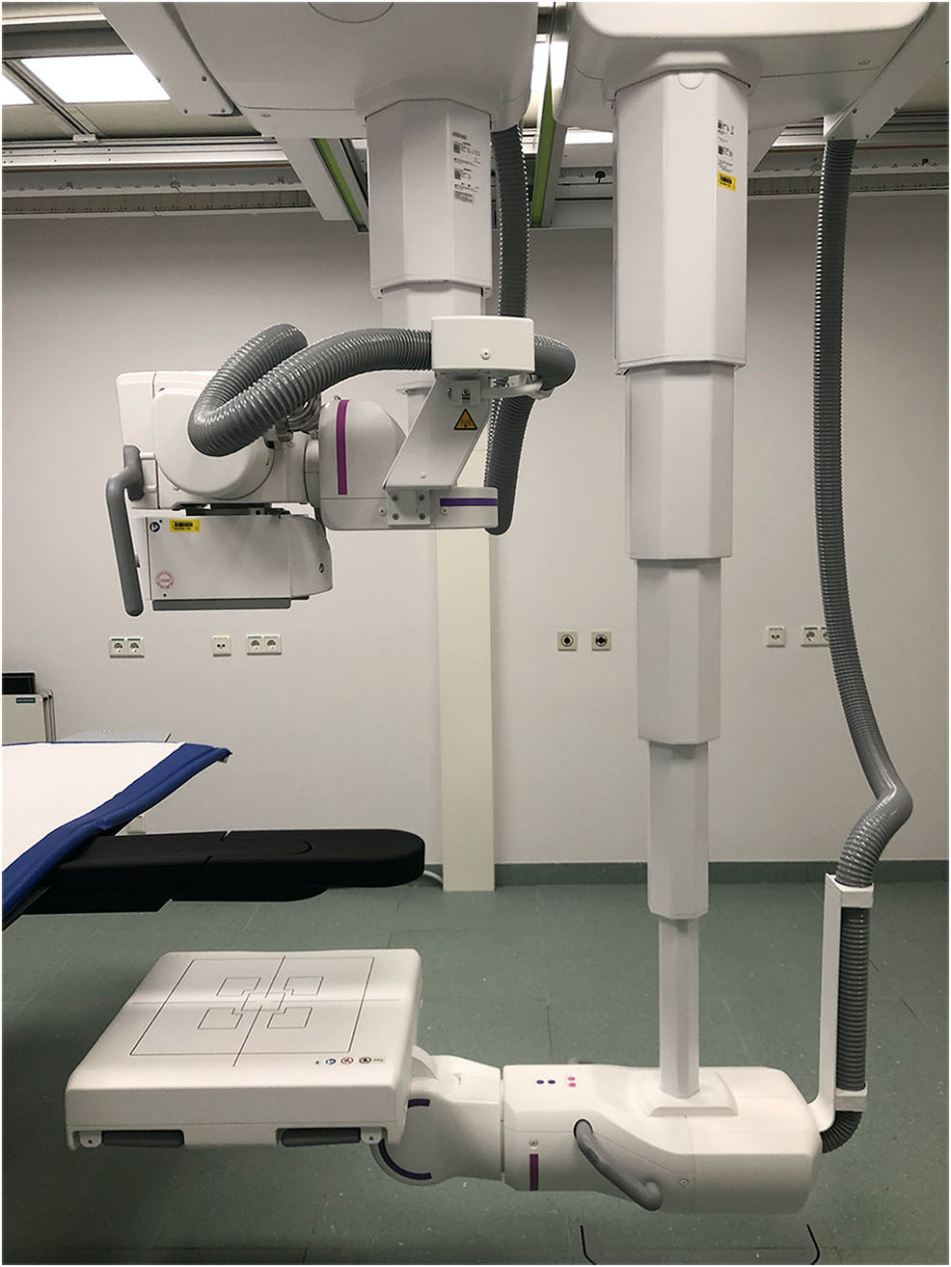
Display of the tableside scan position for wrist imaging with the twin robotic x-ray system

All radiographical and cone-beam CT examinations were performed by trained radiographers with the same twin robotic x-ray system (Multitom Rax, Siemens Healthineers). The multi-use scanner possesses two telescopic arms mounted on ceiling rails that carry the x-ray tube and flat-panel detector (Fig. 2). Individual arm movement is used for 2-D imaging, while simultaneous movement along pre-defined trajectories enables recording of 3-D projection images in CBCT scan mode.

Digital radiographs were acquired first using the clinical standard scan protocol in anteroposterior (AP) and lateral views. For dedicated imaging of the scaphoid bone, additional scans in ulnar deviation with internal or external rotation were conducted. Foot imaging included ancillary oblique projections. Median tube voltage was 50 kV for AP and 55 kV for lateral views, while tube current was 2.0 and 2.2 mAs. For comparison, dose-area products (DAP) were recorded and effective dose (ED) values were calculated with standardized DAP-to-ED conversion factors [24].

After x-ray analysis by radiologists, CBCT examinations were carried out with the same multi-use system as radiographic scans with patients in a supine position and the afflicted extremity in the iso-center of the tube-detector rotation. For wrist, hand, and finger imaging, the upper extremity was positioned besides the patient table, while foot and ankle scans were performed with the concerned leg in extension and the contralateral leg bent to remain outside of the FOV. Scan time was 20 s for all CBCT studies. Acquiring 16 frames per second with the current commercially available software version (VF10, Siemens Healthineers) and considering the acceleration and deceleration phase at the start and end of scans, 298 projection images were recorded per scan. The flat-panel detector provides an image matrix of 1420 × 1436 pixels with 0.3 mm pixel size after 2 × 2 binning. Tube potential of 78.9 kV and copper pre-filtration with thickness of 0.3 mm was in effect for all scans. With automatic dose modulation being active and using our standard clinical scan protocol, the median tube-current-time-product per scan was 114.7 mAs (interquartile range [IQR] 98.1–132.3 mAs). Comparable to x-ray scans, DAP was recorded automatically by the scanner during image acquisition with subsequent calculation of ED values [25]. Scan parameters and radiation dose of radiography and CBCT examinations are depicted in Table 1.

**Table 1:** Scan parameters. Acquisition parameters and radiation dose for cone-beam CT and radiography using the twin robotic x-ray system

CBCT	3-D scan	Radiography	AP/lateral view
Tube voltage (kV)	78.9	Tube voltage (kV)	50/55
Median tube current (IQR) (mAs)	114.7 (98.1–132.3)	Tube current (mAs)	2.0/2.2
Median DAP (IQR) (dGy·cm^2^)	17.5 (13.6–21.8)	Median DAP (IQR) (dGy·cm^2^)	0.2 (0.1–0.3)
Median ED (IQR) (μSv)	4.3 (3.3–5.3)	Median ED (IQR) (μSv)	0.2 (0.1–0.3)

*AP*, anteroposterior; *CBCT*, cone-beam CT; *DAP*, dose-area product; *ED*, effective dose; *IQR*, interquartile range

Reconstruction of projection images was conducted with a dedicated bone kernel in axial, coronal, and sagittal planes. Reconstruction parameters for CBCT scans included slice thickness of 2.0 mm and increment of 1.0 mm. A FOV of 80.0 mm was chosen for upper extremity imaging, while lower extremity scans were reconstructed with a FOV of 100.0 mm for all reformats. Standard settings for window width and center were 3000.0 and 1000.0 Hounsfield units, respectively.

### Image analysis

In order to provide a realistic clinical scenario, primary evaluation of radiographic and CBCT images was performed by on-call radiology residents with 1 to 5 years of clinical experience. All reports received supervision by board-certified radiologists with 5 to 18 years of experience before consensually determining the final diagnosis. While reading CBCT studies, residents and supervisors had access to the previously acquired radiographic scans. They were not aware of the detailed results of the physical examination, though. Dedicated picture archiving and communication system software (Merlin, Phönix-PACS) was used. Detection of acute fractures, articular involvement, and multi-fragment situations was compared between the reports of x-ray and CBCT scans. If more than one fracture was present in the same anatomical region (upper extremity regions: distal forearm, scaphoid, other carpal bone, metacarpal, finger; lower extremity regions: ankle, calcaneus, other tarsal, metatarsal, toe), the injury was counted as one multi-fragment fracture. No patients displayed fractures in more than one of the defined regions. In addition to assessing the diagnosis, residents and supervisors were requested to state their diagnostic confidence using a 5-point scale in consensus reading (5 = total confidence; 4 = high confidence; 3 = moderate confidence; 2 = little confidence; 1 = no confidence). The presence of motion artifacts in CBCT was also evaluated subjectively on a 5-point scale (5 = no artifacts, 4 = slight artifacts, 3 = moderate artifacts, 2 = considerable artifacts, 1 = strong artifacts).

Surgical reports and clinical follow-up of at least 4 weeks were available in 52 and 85 patients, respectively, serving as the standard of reference. Follow-up included clinical examinations as well as repeated and/or additional imaging. Seven patients with negative fracture assessment in x-ray and CBCT scans did not return for clinical follow-up. One patient with inconspicuous imaging received an additional MRI after 1 week for persisting wrist pain, revealing a previously undetected scaphoid fracture.

To evaluate the actual impact of CBCT on treatment decisions, an independent orthopedic surgeon with 5 years of clinical experience retrospectively reviewed all radiological reports. The surgeon was blinded to any additional information (actual therapy, clinical follow-up) and tasked to state the recommended treatment before and after receiving the ancillary information provided by CBCT. For therapy recommendations, the surgeon could choose between no treatment, immobilization/functional treatment, and surgery.

### Statistics

Statistical analysis was performed using specialized software (SPSS Statistics Version 27 for Mac, IBM). Continuous variables were tested for normal distribution using the Kolmogorov-Smirnov test [26]. Normally distributed variables are displayed as means ± standard deviation, whereas categorical variables are presented as frequencies and percentages with median values and interquartile ranges. Wilcoxon signed-rank tests were applied for comparing continuous non-parametric paired variables, while the Mann-Whitney *U* test was exercised to compare non-parametric unpaired variables [27, 28]. Comparison of paired dichotomous data was conducted with the McNemar test [29]. To determine agreement between therapy recommendations of the orthopedic surgeon and actual therapy, Cohen’s weighted kappa was calculated and interpreted according to Landis and Koch (1.00–0.81 = almost perfect agreement; 0.80–0.61 = substantial agreement; 0.60–0.41 = moderate agreement; 0.40–0.21 = fair agreement; 0.20–0.00 slight agreement; < 0.00 poor agreement) [30, 31]. Statistical significance is indicated by *p* values ≤ 0.05.

## Results

**Fig. 3:**
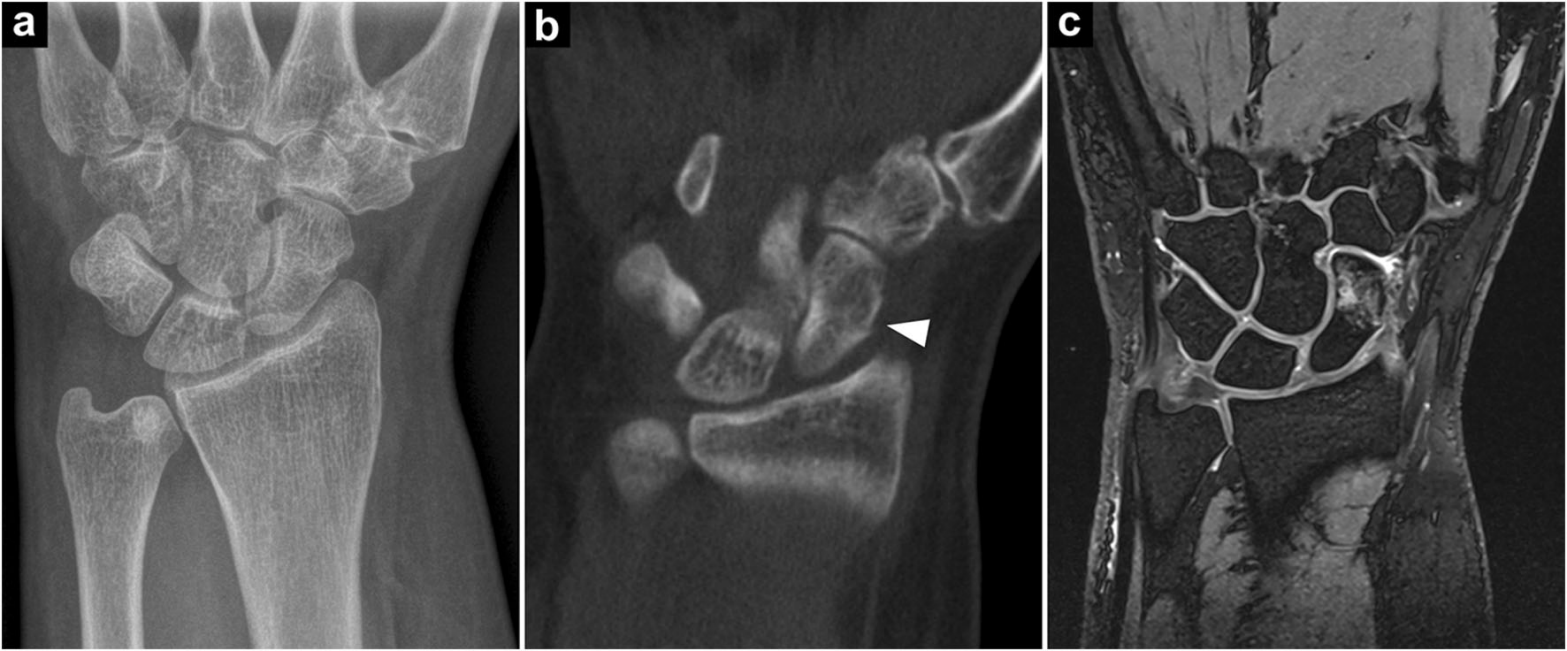
A 55-year-old woman presented to the emergency department with radial-sided left wrist pain and swelling after slipping during yoga class. Initial radiography was deemed negative for fracture presence (**a**). Subsequent cone-beam CT demonstrated a subtle fracture line in the waist of the scaphoid bone (arrowhead), corresponding to a potentially unstable B2 fracture (**b**). Suggested treatment was upgraded to surgery; however, the patient decided for conservative therapy. MRI follow-up after 1 week confirmed the fracture diagnosis, depicting extensive bone bruise in the distal portion of the scaphoid (**c**)

**Fig. 4:**
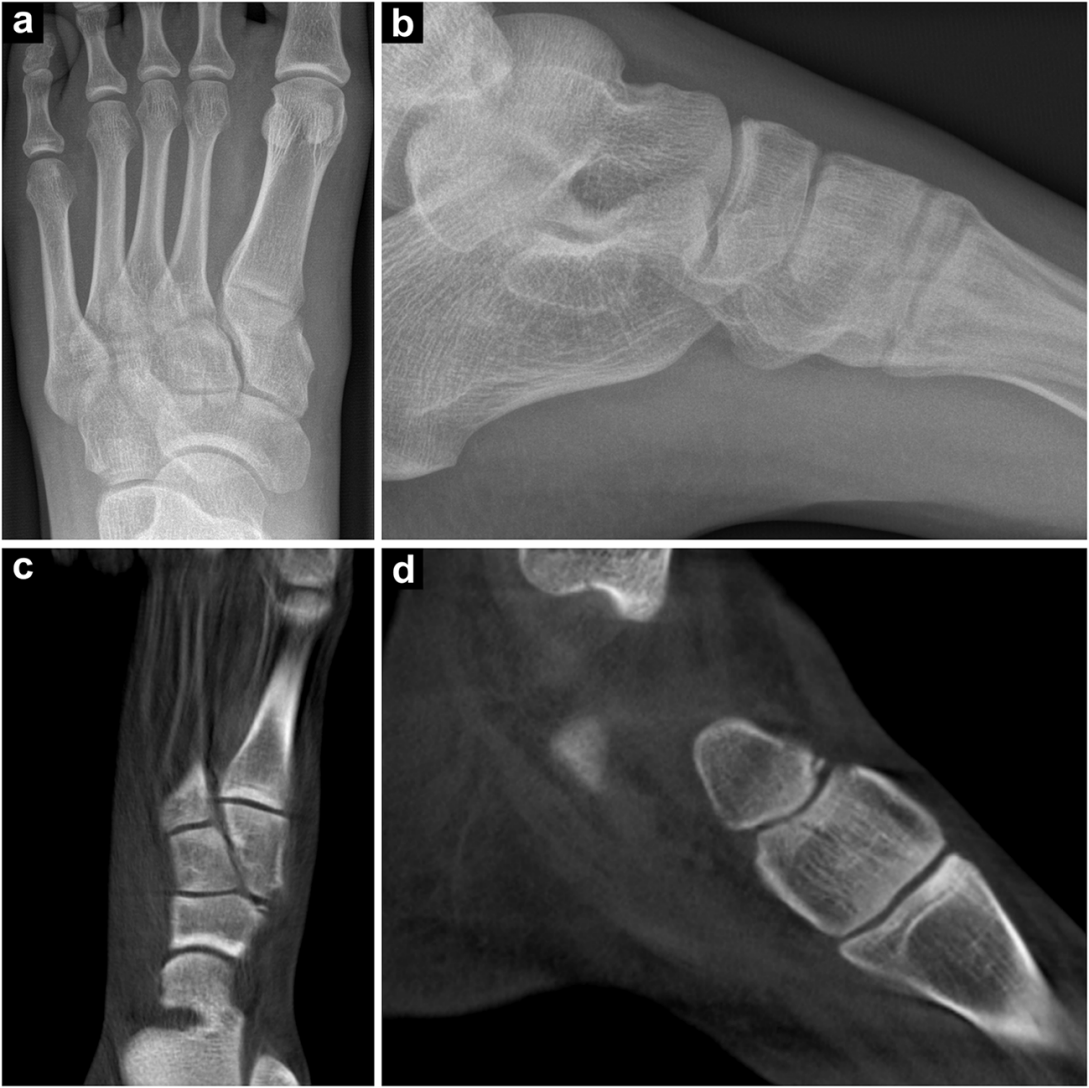
A 22-year-old male sprinter was admitted with increasing left ankle pain after an acute sprain in training. He was unable to bear weight on the left foot and stated pressure pain over the proximal first metatarsal. No fracture could be located in anteroposterior and lateral x-ray scans (**a**, **b**), whereas ensuing cone-beam CT depicted a medial avulsion fracture of the left navicular bone (**c**, **d**). Treatment was upgraded to cast immobilization

**Fig. 5:**
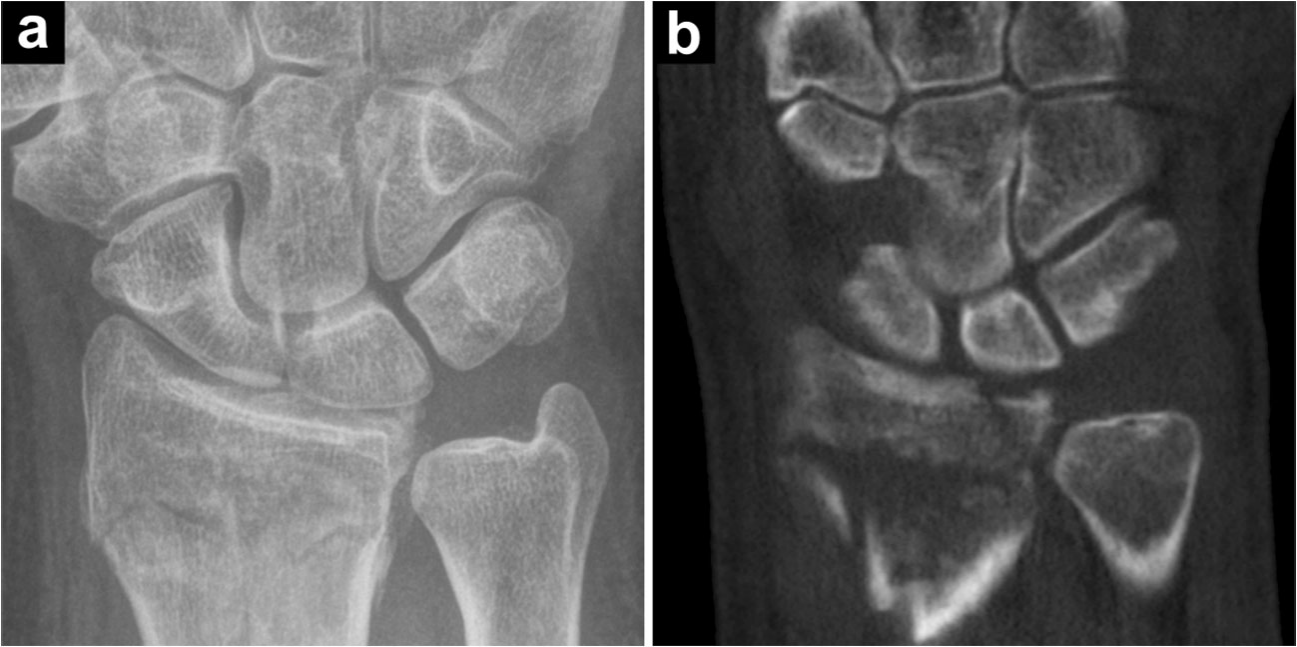
After a fall on the outstretched hand, x-rays of a 63-year-old man exhibited a distal radius fracture with metaphyseal impaction (**a**). While articular surface affliction could not be ascertained in radiography, the cone-beam CT scan demonstrated involvement of the radiocarpal and distal radioulnar joint (**b**). Open reduction was advised irrespective of CT imaging and the reported impaction of the lunate fossa was corrected intraoperatively

**Fig. 6:**
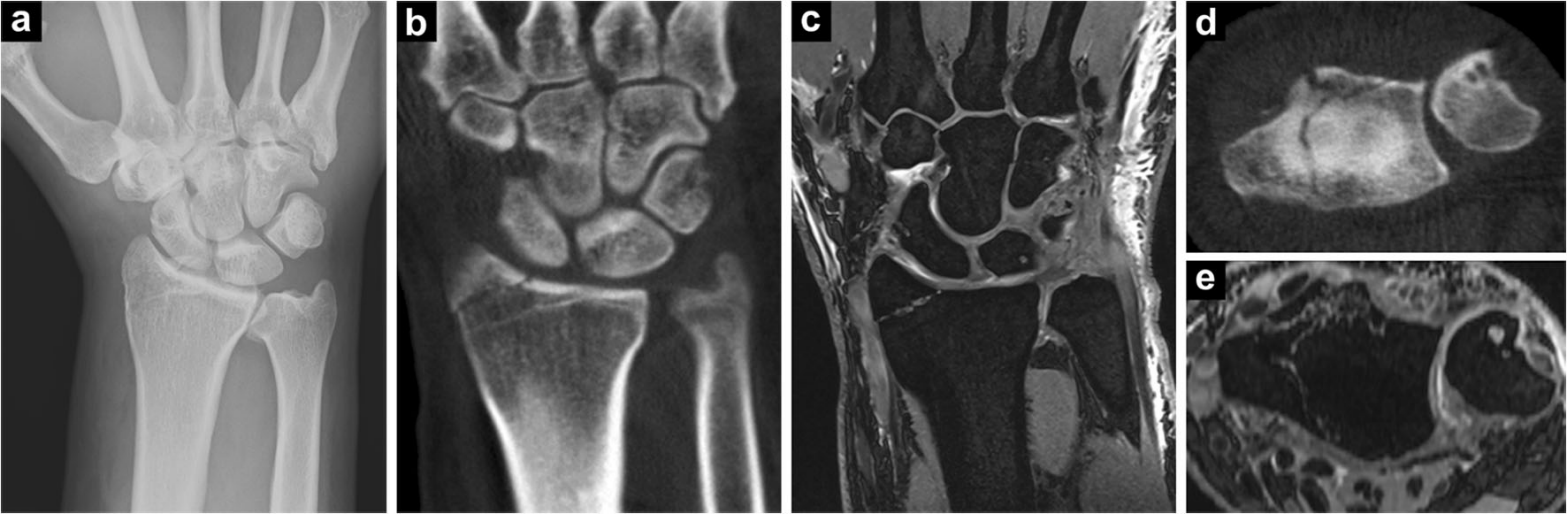
While unloading a truck, a 51-year-old man fell from approximately 2 m height onto his head and right hip. Also reporting right wrist pain, a simple non-displaced fracture of the radial styloid was surmised after radiography (**a**). Coronal reformatting of subsequent cone-beam CT (**b**) confirmed the suspected injury, whereas axial view (**d**) disclosed additional fracture lines in the articular surface of the distal radius. Coronal and axial multiplanar reconstructions of 3-D dual-echo steady-state MRI (**c**, **e**) validated cone-beam CT findings. Treatment was upgraded from immobilization to surgery

In 92 patients examined after acute trauma, 83 out of 85 fractures of the appendicular skeleton were detected with CBCT compared to 64 fractures in digital radiography (accuracy 0.98 vs. 0.71; *p < 0.001*). Upper extremity fractures were diagnosed in 69 CBCT and 53 x-ray examinations, while lower extremity fractures were identified in 14 CBCT and 11 x-ray scans. Seven patients (8.2%) did not present any fractures or fracture-related findings. Of the 20 fractures that were solely detected in CBCT (23.5% of all fractures), 16 concerned the upper extremity, while four involved the lower extremity (Figs. 3 and 4). One toe fracture diagnosed in radiography could not be confirmed in CBCT due to severe motion artifacts. In six patients (6.5%), CBCT ruled out fractures that were suspected in x-ray scans. Affliction of an articular surface was ascertained in 69 of 71 cases with CBCT compared to 53 detected articular fractures in radiography (accuracy 0.98 vs. 0.79; *p < 0.001*) (Fig. 5). Articular fractures of the upper extremity were detected in 57 (CBCT) and 45 (x-ray) of 58 cases, whereas joint involvement of the lower extremity was detected in 12 (CBCT) and 8 (x-ray) of 13 articular fractures. Furthermore, CBCT scans provided superior detection of multi-fragment situations, detecting 68 of 70 comminuted fractures compared to 50 in radiography (accuracy 0.98 vs. 0.76; *p > 0.001*) (Fig. 6). This included upper extremity (55/41 of 56) and lower extremity trauma (13/9 of 14). Detection of fractures and fracture-related findings in CBCT and radiography is summarized in Table 2 (upper extremity) and Table 3 (lower extremity).

**Table 2:** Detected fractures and fracture-related findings in upper extremity trauma

	Cone-beam CT				Radiography			
	TP	FP	FN	TN	TP	FP	FN	TN
Upper extremity fractures	69	0	1	6	53	5	17	1
Articular involvement	57	0	1	18	45	1	13	17
Multi-fragment fractures	55	0	1	20	41	2	15	18
Distal forearm fractures	39	0	0	0	36	0	3	0
Articular involvement	37	0	0	2	34	1	3	1
Multi-fragment injury	37	0	0	2	33	0	4	2
Scaphoid fractures	8	0	1	5	2	4	7	1
Articular involvement	4	0	1	9	1	0	4	9
Multi-fragment injury	4	0	1	9	0	0	5	9
Other carpal fractures	7	0	0	1	3	1	4	0
Articular involvement	3	0	0	5	0	0	3	5
Multi-fragment injury	2	0	0	6	0	2	2	4
Metacarpal fractures	9	0	0	0	8	0	1	0
Articular involvement	8	0	0	1	6	0	2	1
Multi-fragment injury	9	0	0	0	5	0	4	0
Finger fractures	6	0	0	0	4	0	2	0
Articular involvement	5	0	0	1	4	0	1	1
Multi-fragment injury	3	0	0	3	3	0	0	3

*TP*, true positive; *FP*, false positive; *FN*, false negative; *TN*, true negative

**Table 3:** Detected fractures and fracture-related findings in lower extremity trauma

	Cone-beam CT				Radiography			
	TP	FP	FN	TN	TP	FP	FN	TN
Lower extremity fractures	14	0	1	1	11	1	4	0
Articular involvement	12	0	1	3	8	0	5	3
Multi-fragment fractures	13	0	1	2	9	0	5	2
Ankle fractures	7	0	0	0	7	0	0	0
Articular involvement	7	0	0	0	6	0	1	0
Multi-fragment injury	7	0	0	0	5	0	2	0
Calcaneus fractures	2	0	0	0	2	0	0	0
Articular involvement	2	0	0	0	1	0	1	0
Multi-fragment injury	2	0	0	0	2	0	0	0
Other tarsal fractures	3	0	0	0	0	0	3	0
Articular involvement	2	0	0	1	0	0	2	1
Multi-fragment injury	2	0	0	1	0	0	2	1
Metatarsal fractures	1	0	0	1	1	1	0	0
Articular involvement	0	0	0	2	0	0	0	2
Multi-fragment injury	1	0	0	1	1	0	0	1
Toe fractures	1	0	1	0	1	0	1	0
Articular involvement	1	0	1	0	1	0	1	0
Multi-fragment injury	1	0	1	0	1	0	1	0

*TP*, true positive; *FP*, false positive; *FN*, false negative; *TN*, true negative

Fifty-two patients (56.5%) received surgical therapy after imaging (44 upper/8 lower extremities), with the majority of surgical procedures (34) comprising open reduction and internal fixation of distal forearm fractures. Thirty-four patients (37.0%) were treated solely with immobilization or functional therapy (27/7). Treatment change based on additional findings in CBCT was recommended in 29 patients (24/5) with upgrades and downgrades of therapy suggested in 23 (19/4) and 6 (5/1) patients. Most upgrades were from no therapy to immobilization or functional treatment due to detection of radiographically occult fractures in CBCT (15/4). Three previously undetected scaphoid fractures were upgraded to surgery. All six downgrades were recommended because CBCT ruled out fractures previously suspected in x-ray scans, including five presumed carpal and one metatarsal fracture. Agreement between recommendations after CBCT imaging and actual treatment was almost perfect (*κ* = 0.88 [95% confidence interval 0.83–0.93]; *p < 0.001*), whereas agreement between suggestions before CBCT scans and therapy was moderate (*κ* = 0.41 [0.35–0.47]; *p < 0.001*). Detailed treatment suggestions are depicted in Table 4.

**Table 4:** Treatment recommendations before and after cone-beam CT

Suggestion before cone-beam CT	Suggestion after cone-beam CT	Overall	Upper extremity	Lower extremity
No therapy	No therapy	2	2	0
	Immobilization/functional treatment	19	15	4
	Surgery	3	3	0
Immobilization/functional treatment	No therapy	4	3	1
	Immobilization/functional treatment	11	9	2
	Surgery	1	1	0
Surgery	No therapy	2	2	0
	Immobilization/functional treatment	0	0	0
	Surgery	50	41	9

Regarding the perceived effect of patient movement on CBCT image quality, no motion artifacts (score 5) were reported in 67 of 92 examinations (72.8%). In upper extremity imaging, slight motion artifacts (score 4) were present in 14 of 76 patients (18.4%), while lower extremity CBCT studies exhibited at least moderate artifacts (scores 1–3) in 6 of 16 studies (37.5%). Overall, motion artifacts were stronger in lower extremity imaging (*p < 0.001*). Diagnostic confidence of radiologists was superior for CBCT examinations compared to radiography (*p < 0.001*). Total diagnostic confidence (scale value 5) was declared in 89 of 92 CBCT (96.7%) and 41 of 92 x-ray scans (44.6%). Little to no confidence (scale values 1 and 2) was reported in 12 radiographic examinations (13.0%) and one CBCT scan (1.1%). Detailed ratings for diagnostic confidence are shown in Table 5.

**Table 5:** Diagnostic confidence

	Overall		Upper extremity		Lower extremity	
Confidence	Radiography	Cone-beam CT	Radiography	Cone-beam CT	Radiography	Cone-beam CT
5	41 (44.6)	89 (96.7)	35 (46.1)	75 (98.7)	6 (37.5)	14 (87.5)
4	18 (19.6)	2 (2.1)	15 (19.7)	1 (1.3)	3 (18.8)	1 (6.3)
3	21 (22.8)	0 (0.0)	16 (21.1)	0 (0.0)	5 (31.3)	0 (0.0)
2	8 (8.7)	0 (0.0)	7 (9.2)	0 (0.0)	1 (6.3)	0 (0.0)
1	4 (4.3)	1 (1.1)	3 (3.9)	0 (0.0)	1 (6.3)	1 (6.3)
Median	4.0	5.0	4.0	5.0	4.0	5.0

Median DAP for CBCT scans was 17.5 dGy·cm^2^ (IQR 13.6–21.8 dGy·cm^2^), corresponding to an ED of 4.3 μSv (3.3–5.3 μSv). For radiography, the median DAP and ED of the AP and lateral view combined were 0.2 dGy·cm^2^ (0.1–0.3 dGy·cm^2^) and 0.2 μSv (0.1–0.3 μSv), respectively.

## Discussion

### Main findings

We evaluated the detection of fractures and fracture-related findings in small bone and joint trauma using the radiography and CBCT functions of a twin robotic, multi-purpose x-ray system. Focusing on the clinical importance of additional 3-D imaging, treatment recommendations based on image findings before and after CBCT were compared. With the CBCT scan mode, presence of fractures, articular joint involvement, and multi-fragment situations were correctly diagnosed more often than with conventional x-ray scans. CBCT imaging also facilitated ruling out several fractures suspected in radiography that would have led to unnecessary immobilization. Falkowski et al recently compared trauma assessment with MDCT and CBCT, showing good results for fracture detection and image quality with an earlier version of the CBCT scan mode [23]. We assume, however, that the added clinical value of CBCT scans lies mostly in the supplemental information provided over radiography and the influence of these findings on therapeutic decision-making. In our study, change of treatment after CBCT scans was suggested in approximately one third of patients. This is in line with the results of Brink et al who analyzed fracture detection between single-shot MDCT and radiographs [7], but at the same time is almost twice the percentage of therapy change in another recent study by Alagic et al [11]. Unlike the mentioned studies, the incidence of surgical treatment was considerably higher in our work. However, even though more than 50% of patients underwent surgery, only three acute fractures that needed operative treatment were not detected in x-ray scans (all concerning the scaphoid bone). In contrast, two suspected scaphoid waist fractures were ruled out by CBCT, preventing unneeded surgical intervention. Our results show that, apart from surgical planning, the addition of CBCT scans is especially helpful for diagnosing and excluding occult or subtle fractures of carpal and tarsal bones. Being a common reason for carpal instability and therefore oftentimes requiring surgery, especially scaphoid fracture assessment benefitted from the ancillary 3-D imaging. While a notable increase in diagnostic confidence was expected, the vast majority of absolute confidence ratings after CBCT might improve the clarity of radiological reports and subsequently the quality of interdisciplinary communication in trauma assessment. It is important to note that CBCT studies feature characteristic image traits that may at first be unfamiliar to colleagues only used to MDCT studies (e.g., cone-beam artifacts, decreased soft tissue contrast). In order to accustom orthopedic surgeons to the distinct image appearance of CBCT scans, we suggest close collaboration in clinical routine.

Effective radiation dose for CT imaging tasks of the appendicular skeleton is generally lower than for other body regions due to the small tissue-weighting factor. Nonetheless, the tested system possesses potential for dose reduction compared to state-of-the-art MDCT [21, 22]. Whereas reported radiation dose for MDCT scans in small bone and joint trauma ranges from 10 to 800 μSv [10, 14, 32, 33], a meta-analysis by Nardi et al stated an average effective dose of 7.1 μSv for CBCT examinations of extremities [34]. Corresponding to only 12 h of exposure to background radiation [35], our median effective dose of 4.3 μSv is far below the current literature average but still above the reference values for conventional x-ray imaging. Future studies should aim to reduce radiation dose in extremity CBCT even further to achieve comparable values to radiography.

Despite the long acquisition time of 20 s, motion artifacts were less common in CBCT scans than expected. However, a mismatch could be observed between body regions, with wrist/hand/finger studies exhibiting substantially less artifacts than ankle/foot/toe examinations. This may be due to superior positioning options for upper extremity imaging provided by the two telescopic arms of the multi-use x-ray system. The tableside trajectory allows for patients to be examined in a supine position with the injured arm abducted by 90°. Especially for patients with elbow or shoulder comorbidities, empirical knowledge shows that this position is perceived as more comfortable than the “superman position” necessary for MDCT studies [36]. In contrast, lower extremity projection images were acquired with the afflicted leg extended and the opposite leg bent to not interfere with the FOV. The imbalance of this position might have led to increased patient movement in some cases. One foot study of a patient with slight tremor was deemed not diagnostic, suggesting that assessment of the patients’ ability to lie still should always precede CBCT imaging.

With recent studies on dual-energy CT (DECT) also achieving promising results in the detection of radiographically occult fractures [37, 38], a direct comparison of the two techniques in emergency settings should be the scope of future research. Using virtual non-calcium imaging, DECT has the ability to delineate bone marrow edema in cancellous bone. Acquisition time is short and DECT imaging is not limited to the appendicular skeleton due to larger scan volumes and superior soft tissue contrast. For CBCT, in addition to superior spatial resolution, weight-bearing imaging can provide ancillary diagnostic information with regard to joint stability [20, 39]. Concerning workflow, the novel system provides the combination of radiography, fluoroscopy, and CBCT within the same examination room, rendering repeated positioning and additional patient transport between x-ray suite and CT unnecessary.

### Limitations

Several limitations have to be acknowledged for this study. Scans were performed in a realistic clinical setting at a radiology department of a university hospital. Therefore, the patient group was heterogeneous regarding sociodemographic data, localization of injury, and severity of trauma. On-call staff and supervisors with different levels of clinical experience performed the reporting. Using consensus decisions of residents and supervisors for diagnosis and confidence ratings, the junior radiologists’ opinion might have been overshadowed by their supervisors’ assessment with particular bias concerning the confidence estimation. As CBCT scans are generally conducted if x-ray imaging does not provide sufficient information to decide on diagnosis and treatment, radiologists were not blinded to the results of previous radiographs when evaluating the CBCT images. Wrist, hand, and finger imaging tasks (82.6%) were more common than ankle, foot, and toe scans (17.4%). Due to the CBCT scan mode’s narrow FOV, large joints and bones, such as hips, shoulders, and the vertebral column, were not assessed in this study. No weight-bearing imaging was conducted. As CBCT is known to be particularly suitable for visualization of dense structures (e.g., bone, teeth), this work was focused on fractures and fracture-related findings. Even though non-fracture-related findings and soft tissue injuries were assessed by the radiologists, they did not factor into the study analysis. With Siemens Healthineers Multitom Rax and VF10 not being available in all countries, reproducibility of results may be limited.

## Conclusion

Cone-beam computed tomography provides a substantial added value for the assessment of small bone and joint trauma by detecting and excluding extremity fractures and fracture-related findings more reliably than conventional radiography. Additional information led to a change of treatment in one third of patients, suggesting a significant clinical impact. Diagnostic confidence improved considerably compared to x-ray scans. Being able to acquire radiographs and CBCT data in the same position with the multi-use scanner facilitates one-stop-shop imaging in acute trauma of the peripheral skeleton.

## Abbreviations

CBCT: Cone-beam computed tomography
DAP: Dose-area product
DLP: Dose-length product
ED: Effective dose
FOV: Field of view
IQR: Interquartile range
